# Dr. Spark on the Use of Opium in Spasm of the Uterus

**Published:** 1813-07

**Authors:** William Spark

**Affiliations:** Ipswich


*To the Editors of the Medical and Physical Journal.*

GENTLEMEN,

DURING a long and extensive course of practice in Midwifery, like other practitioners, I have been subject to very tedious and vexatious labours, occasioned by a spasmodic affection of the uterus, which I found very difficult to remove, and until removed true labour pains were suspended. This spasmodic obtrusion would sometimes continue many hours, and even days; the suffering women were worn down by fatigue, and after parturition they recovered very slowly. After being teased with these cases nearly forty years, a case of obstructed intestine from spasm came under my care, where no time was to be lost; life or death depended upon immediate and bold effort. The patient, a female, had been attended by a practitioner very little in the habit of prescribing medicines, about fifty hours. She brought up all the contents of the stomach and bowels above the stricture, no evacuation below could be procured, and and gangrene was expected. I gave her six grains of solid opium, and half a drachm of pil. ex colocynth. c̄u al. the first to remove spasm, and the latter to empty the canal immediately afterwards, to prevent a return. On visiting her two hours after taking the pills, I found the opium was not in sufficient dose, and gave her two grains more: this relieved the obstruction, the colocynth pill immediately acted, and the patient recovered. It may be proper to remark in this case, that the first medical friend gave his medicines in a liquid form, which the irritable stomach rejected; I gave mine in a solid form, and they remained. From the happy termination of this case, it occurred to me, that if a large dose of opium was necessary to remove the spasm, it was equally so in other cases, though the cause was different. I began to give it cautiously, in doses of three, four, or five, grains, in spasmodic affections of the uterus, and with some advantage; no ill effect arising, I grew bolder, and gave it frequently in doses of six grains, sometimes eight, and in one case ten; the consequences have been most happy to my patients, and a great relief to myself; my general dose has been from six to eight grains, according to the degree of spasm. I have exhibited these doses in more than a hundred cases, without any particular inconvenience, except in a few instances the head has been slightly affected, similar to intoxication; in others the stomach, where the irritation did not reach it to occasion sickness, the opium did, but where the stomach was irritated to sickness, the opium removed it. These effects were of short duration, and bear no comparison to the benefits received: the spirits of the women are exhilarated, the uterus performs its functions with vigor, it gives way rapidly to the pressure of the child, the placenta never adheres, hemorrhages never follow, the uterus retains nothing, of course the patient is not afflicted with after-pains, and she recovers her strength and health more quickly than those who need not the aid of opium. Independent of the above advantages, I never saw milk or puerperal fever where opium had been given.

What I call spasmodic pains of the uterus, are those generally termed spurious, or cholic. The patients most subject to them are weakly, or what are said to be nervous. The general symptoms are a frequent desire to void urine in small quantities, the os uteri little dilated, and during pain instead of dilating it contracts, and the spirits of the women are depressed.

To the above remarks I will add a few cases:—

Mrs. B. a merchant's lady, found the effects of opium so powerfully powerfully in her third labour, as to induce her to send to me in the beginning of her fourth for the same medicine.

Mrs. R. a merchant's lady, suffered many hours with spurious pains. I wished her to take some pills of opium; she replied, that she never had taken a pill, and believed she could not swallow one. I then gave her sixty drops of laudanum, and waited two hours without its having any good effect; I prevailed on her to try and take two pills, which she swallowed with difficulty; they contained six grains of opium. In about half or three quarters of an hour, she said she was relieved; and had she known the effect the pills would have, she would have taken them instead of the drops. Her labour now went on quick.

Mrs. F. inclined to labour, but spurious pains suspended it. I gave her ten grains of opium, which removed the spasms, but pains did not follow. She appeared languid, from a larger dose of opium than was necessary. I thought some warm stimulus would relieve, and gave her a dish of tea; it was no sooner in the stomach than pains came on, and she was delivered in about ten minutes.

Mrs. L. with her first child suffered constant pains for forty-eight hours. She was attended by a female practitioner, whose education was of the best kind, and who was in constant expectation of delivery: both midwife and patient were worn out; the former wished to be relieved by another female practitioner, but Mrs. B., with whom she had lived as a servant, desired my attendance. I found her suffering with spurious pains, and the midwife informed me that she had been in exactly the same state for forty-eight hours. I endeavored to give an enema without effect; two hours were lost, and she remained the same: I then gave her six grains of opium, and told her she would find the pains alter in about half or three quarters of an hour. About that time she said, "the medicine is at work, and I do not mind these pains." Labour now went on well, and she was delivered within the hour and half from the time of taking the opium.

Mrs. C., in her three first labours, suffered from spurious pains about a week, and she was supposed to be in labour all the time. Her practitioner was not deficient in skill, but not in the habit of giving opium. She had heard of my success, and requested I would attend her in the fourth, which I did, and she had just such a tedious time as before. I would not give opium, because I wished to watch the case, to enable me to draw a fair conclusion from the same patient between opium omitted and opium given. In her fifth labour I was sent for to her and another woman at the same time;

 both both suffered from spasms; I gave to each six grains of opium; both were relieved, labour pains succeeded the spurious, and they were delivered within two hours.

Mrs. J., fifteen years of age, in labour of her first child, sent for me about five o'clock in the morning. The os uteri was not dilated, and the thickest I ever felt, her pains spurious. This case appeared to me to require a full dose of opium, and I gave her eight grains. At ten o'clock I was sent for again, the spasms were removed, the uterus giving way, and she was delivered at half past eleven. This case appeared to me likely to be very tedious.

Mrs. D., attended by a medical friend, between seven and eight months advanced in pregnancy, had been subject to uterine hemorrhage three weeks, and brought to that state as to make delivery necessary. I was requested to attend. The body of the placenta was immediately over the os uteri. I advised five grains of opium to be given immediately, and to deliver by turning the child an hour afterwards, when the uterus was under the influence of the opium, and would not resist. I attended again at that time; the hemorrhage was abated, I advised immediate delivery; her medical friend was timid, and requested me to act. I endeavored to separate the placenta from the uterus, but found difficulty in doing it. I then passed my hand through the body of the placenta near the funis up to the fundus uteri, took the feet, and delivered; the placenta was brought away soon, and the whole accomplished without the loss of more blood than is usual in common labours. Mrs. D. was well enough to sit up the third day and nurse her child; she recovered rapidly.

Uterine hemorrhages preceding abortion, which so frequently happen when women are advanced about three or five months in pregnancy, may be much relieved, if not totally removed, by a proper dose of opium, assisted by rest, and the horizontal position.

The practitioner ought to distinguish between the tedious cases arising from spasmodic affection of the uterus, and the laborious cases arising from rigid muscular fibre in strong young women, or those advanced in years, with first children. In the former, opium has the happiest effects; in the latter, I suppose, none. The tincture of opium has not the effect in any dose. I have selected the best crude opium in preference to the strained, which I thought affected the stomach more than the crude, from what cause I cannot account, unless the action of fire injured its quality.

The above remarks and cases were not intended for the public eye; I made no minutes of them at the time, and write from memory; they are published at the particular request request of several very eminent medical gentlemen. I have no interest beyond the pleasure of being the cause of relief to the fair sex, and the anxious time saved to my professional brethren, having declined the practice several years, and become a member of an university. I have endeavored to be concise, only stating matters of fact; if I have not explained myself sufficiently, I shall be ready to give candid answers to any queries; and I hope when any practitioner has given this mode of relief a fair trial, he will give publicity to his observations. I am, &c.

*Ipswich.* WILLIAM SPARK, M.D.

